# Cyclophilin A enhances cell proliferation and tumor growth of liver fluke-associated cholangiocarcinoma

**DOI:** 10.1186/1476-4598-10-102

**Published:** 2011-08-26

**Authors:** Sumalee Obchoei, Sarah M Weakley, Sopit Wongkham, Chaisiri Wongkham, Kanlayanee Sawanyawisuth, Qizhi Yao, Changyi Chen

**Affiliations:** 1 Molecular Surgeon Research Center, Michael E. DeBakey Department of Surgery, Baylor College of Medicine, Houston, Texas, USA; 2 Department of Biochemistry, Liver Fluke and Cholangiocarcinoma Research Center, Faculty of Medicine, Khon Kaen University, Khon Kaen, Thailand

**Keywords:** cyclophilin A, cholangiocarcinoma, cell proliferation, cyclosporin A, peptidyprolyl *cis-trans *isomerase, ERK1/2 pathway, CD147

## Abstract

**Background:**

Cyclophilin A (CypA) expression is associated with malignant phenotypes in many cancers. However, the role and mechanisms of CypA in liver fluke-associated cholangiocarcinoma (CCA) are not presently known. In this study, we investigated the expression of CypA in CCA tumor tissues and CCA cell lines as well as regulation mechanisms of CypA in tumor growth using CCA cell lines.

**Methods:**

CypA expression was determined by real time RT-PCR, Western blot or immunohistochemistry. CypA silence or overexpression in CCA cells was achieved using gene delivery techniques. Cell proliferation was assessed using MTS assay or Ki-67 staining. The effect of silencing CypA on CCA tumor growth was determined in nude mice. The effect of CypA knockdown on ERK1/2 activation was assessed by Western blot.

**Results:**

CypA was upregulated in 68% of CCA tumor tissues. Silencing CypA significantly suppressed cell proliferation in several CCA cell lines. Likewise, inhibition of CypA peptidyl-prolyl cis-trans isomerase (PPIase) activity using cyclosporin A (CsA) decreased cell proliferation. In contrast, overexpression of CypA resulted in 30% to 35% increases in proliferation of CCA cell lines. Interestingly, neither silence nor overexpression of CypA affected cell proliferation of a non-tumor human cholangiocyte cell line, MMNK1. Suppression of CypA expression attenuated ERK1/2 activity in CCA M139 cells by using both transient and stable knockdown methods. In the *in vivo *study, there was a 43% reduction in weight of tumors derived from CypA-silenced CCA cell lines compared with control vector CCA tumors in mice; these tumors with stable CypA silencing showed a reduced cell proliferation.

**Conclusions:**

CypA is upregulated in majority of CCA patients' tissues and confers a significant growth advantage in CCA cells. Suppression of CypA expression decreases proliferation of CCA cell lines *in vitro *and reduces tumor growth in the nude mouse model. Inhibition of CypA activity also reduces CCA cell proliferation. The ERK1/2 pathway may be involved in the CypA-mediated CCA cell proliferation. Thus, CypA may represent an important new therapeutic target for liver fluke-associated CCA.

## Background

Cholangiocarcinoma (CCA) is a malignant tumor derived from bile duct epithelium. Although it is relatively rare in Europe and North America, CCA occurs at a much higher incidence in Southeast Asia; incidence and mortality rates from CCA are increasing worldwide [1,2]. In the northeast of Thailand, CCA is the most common liver cancer and represents a major health problem in this area. Several clinical risk factors are associated with CCA carcinogenesis. Among these, colonization with the liver fluke *Opisthorchis viverrini *appears to be the most important risk factor for CCA development in this endemic area of Thailand [3].

Clinical treatment options for this cancer are very limited because CCA is refractory to conventional chemotherapy and radiation treatment [4]. At present, complete surgical excision represents the only chance for survival. Unfortunately, distant metastasis, extensive regional lymph node metastasis and vascular invasion frequently preclude resection [5]. Patients with unresectable CCA generally survive fewer than 12 months after diagnosis [6]. It is clear that future research should focus on developing novel chemopreventive and adjuvant therapeutic strategies for CCA; to do this, we have focused on selecting molecular targets that may impact clinical outcomes.

Cyclophilin A (CypA) is an 18 kDa cytosolic protein that is thought to be the major intracellular target of the immunosuppressive drug cyclosporin A (CsA) [7]. CypA possesses a peptidylprolyl *cis-trans *isomerase (PPIase) activity that interconverts the *cis *and *trans *isomers of peptide bonds that precede the amino acid proline [8]. In addition to its enzymatic properties, CypA is conserved throughout the phylogenetic tree from yeast to human; as such, CypA is believed to be a key molecule in many biological functions including molecular chaperoning, protein folding [9], protein trafficking [10], immune modulation [11] and cell signaling [12]. Also, although CypA is present intracellularly, it can be secreted from cells in response to inflammatory stimuli such as hypoxia, infection and oxidative stress [13-16]. A secreted CypA appears to be mediated through its Ig-like membrane receptor, CD147, and stimulate a Ras-dependent extracellular signal-regulated kinase-1 and -2 (ERK1/2) signaling pathway [17,18].

CypA protein has been found to be expressed at unusually high levels in several types of cancers including non-small cell lung cancer, pancreatic adenocarcinoma, hepatocellular carcinoma, oral cancer and buccal squamous cell carcinomas [19-22]. Several studies have shown that CypA promotes cancer cell proliferation, anti-apoptotic phenotype, migration/invasion, and drug resistance in various cancer cell types [23-25]. However, the role of CypA in CCA has not been determined. The purposes of this study were to examine the expression of CypA in CCA tumor specimens and cell lines and to determine the functions and mechanisms of CypA in CCA cell lines *in vitro *and *in vivo*. CypA could be a potential therapeutic target for CCA.

## Methods

### Chemicals and reagents

RPMI 1640 medium, fetal bovine serum (FBS), trypsin EDTA, Opti-MEM I medium and Lipofectamine™ 2000 transfection reagent were purchased from Invitrogen Life Technology. Puromycin and mouse anti-β-actin antibody were purchased from Sigma Chemical Co (St Louis, MO). Rabbit anti-CypA antibody was purchased from Upstate (Charlottesville, VA). Mouse anti-Ki-67 antibody was obtained from Novacastra, United Kingdom. Goat anti-rabbit IgG (H&L) antibody conjugated to horseradish peroxidase (HRP) and goat anti-mouse IgG (H&L) antibody conjugated to HRP were obtained from Cell Signaling Technology Laboratories, Inc. Rabbit anti-ERK1/2 antibody, mouse anti-pERK1/2 antibody, and CsA were obtained from Santa Cruz Biotechnology. All other chemicals were from Sigma.

### CCA tumor specimens and CCA cell lines

Fifty-seven paraffin-embedded histologically proven CCA tumor specimens were obtained from the specimen bank of the Liver Fluke and Cholangiocarcinoma Research Center, Faculty of Medicine, Khon Kaen University, Thailand; patients had undergone liver resection at Srinakarin Hospital, Thailand. Informed consent was obtained from all patients prior to analysis, and the research protocol (#HE471214) was approved by the Human Research Ethics Committee at Khon Kaen University. Specimens were fixed in 10% neutral formalin buffer, embedded in paraffin, and cut into 5 μm thick sections and examined by pathologists for histological confirmation.

Six distinct CCA cell lines, M055, KKU100, M156, M139, M213 and M214, were established from different histological types of primary tumors from patients living in the *Opisthorchis viverrini *endemic area of Northeast Thailand who were admitted for surgical treatment [26]. Cell line M055 and KKU100 originated from patients with poorly differentiated adenocarcinoma. M156 and M214 were derived from patients with moderately differentiated, while M139 and M213 were established from squamous carcinoma and adenosquamous tumor origin, respectively. An immortalized human cholangiocyte cell line, MMNK1, was established as previously described [27] and was used in this study as a non-tumor cell line for comparison. All cell lines were cultured in RPMI 1640 medium with 10% FBS as previously described [21].

### Immunohistochemistry

Tumor specimens from patients with CCA and mouse tumors were fixed overnight in formalin and processed into 5 μm thick sections. The formalin-fixed, paraffin-embedded sections were stained for CypA and/or Ki-67 using standard immunohistochemistry protocols. Briefly, tissue sections were deparaffinized and rehydrated in xylene and ethanol, boiled for 3 minutes in 0.1 M citrate buffer at pH 6.0 to unmask the antigens, and then endogenous peroxidase activity was blocked in a 0.6% hydrogen peroxide (H_2_O_2_) solution. Samples were then blocked for 1 hour, followed by incubation of primary antibody for 1 hour and HRP-conjugated secondary antibody for 1 hour at room temperature. The primary antibody dilutions used were 1:100 rabbit anti-CypA antibody and 1:1,000 mouse anti-Ki-67 antibody. The secondary antibody dilutions used were 1:100 HRP-conjugated goat anti-rabbit IgG and 1:100 HRP-conjugated goat anti-mouse IgG. Sections incubated with phosphate buffered saline (PBS) instead of primary antibody served as negative controls. The sections then underwent treatment with 0.05% 3, 3'-diaminobenzindine tetrahydrochloride (DAB) and 0.1% H_2_O_2 _in 50 mol/L Tris-HCl pH 7.8; and were counterstained with hematoxylin. CypA expression was examined as previously described [28]. Briefly, the frequency of CypA positive cells was semi-quantitatively scored on the basis of the percentage of positive cells, with 0% = negative; 1-25% = +1; 26-50% = +2; and >50% = +3. The intensity of CypA expression was scored as weak = 1, moderate = 2 and strong = 3. The CypA immunoreactivity index of each section was calculated as intensity multiplied by frequency.

### RNA extraction from cells and tissues

Total RNA was extracted using Ambion RNAqueous-4PCR kit (Austin, TX) as previously described [29]. Briefly, cells were lysed using Ambion lysis buffer and then transferred to an Ambion mini-column and centrifuged at 10,000 × *g *for 1 minute. The column was washed 3 times with washing buffer and eluted in 100 μl of elution buffer. RNA solution was treated with DNAse I to remove any trace amounts of genomic DNA contamination, using the Ambion DNA removing kit. For the mouse tumor tissues, the frozen tissues were soaked overnight in RNAlater-ICE buffer (Ambion) before being lysed in Ambion lysis buffer.

### Real-time PCR

CypA mRNA levels were analyzed by real-time reverse transcriptase-PCR as previously described [21]. Briefly, mRNA was reverse-transcribed into cDNA using the iScript cDNA synthesis kit (Bio-Rad). The PCR reaction of 100 nM of each primer, diluted cDNA templates and iQ SYBR Green Supermix, ran for 40 cycles of 95°C for 20 seconds and 60°C for 1 minute. Each cDNA sample was run in triplicate. The β-actin primer was included in every plate to account for sample variations. The mRNA level of each sample was normalized to that of β-actin mRNA. The relative mRNA level was presented as unit values of 2^[Ct(β-actin)-Ct(CypA)]^. The primers for human *CYPA *gene and the housekeeping gene β-actin were designed [21]. The primer sequences for the human *CYPA *gene [GenBank: NM_0211130.3] were 5"GTCAACCCCACCGTGTTCTTC3" (sense) and 5"TTTCTGCTGTCTTTGGGACCTTG3" (antisense), and for the housekeeping gene β-actin, primers were 5"CTGGAACGGTGAAGGTGACA3" (sense) and 5"AAGGGACTTCCTGTAACAATGCA3" (antisense).

### Western blot

Cells were lysed with radio immuno-precipitation assay (RIPA) buffer (Pierce Biotechnology) for 30 minutes in ice. Cell lysates were then collected after centrifugation at 12,000 rpm for 10 minutes at 4°C. Protein lysate (15 μg) was loaded for CypA, ERK1/2, and pERK1/2 protein analysis. Protein bands were separated with 4-20% Tris-Glycine gradient SDS polyacrylamide gel electrophoresis and then transblotted for 1 hour at 4°C onto Hybond-P PVDF membrane (Amersham Biosciences, Arlington Heights, IL). The membrane was probed with rabbit anti-CypA antibody (1:2,000), rabbit anti-ERK1/2 antibody (1:2,000), mouse anti-pERK antibody (1:1,000), or anti-β-actin antibody (1:10,000) at room temperature for 1 hour and washed 3 times with 0.1% Tween 20-Tris-buffered saline (TBS). Then, the membrane was incubated in a HRP-linked secondary antibody (1:20,000) for 1 hour at room temperature and then washed 3 times with 0.3% Tween 20-TBS; the immunoactive bands were detected using an enhanced chemiluminescence (ECL) plus reagent kit.

### Plasmid transfection

All plasmids used in the present study were purchased from OriGene. The effects of CypA silence and overexpression were studied in CCA cell lines and MMNK1. M139, M213, M214, and MMNK1 cells were transiently transfected with HuSH 29mer shRNA construct against CypA (shCypA) to elicit silencing, which was compared to cell lines transfected with the shRNA pRS non-effective GFP plasmids (shV) as a negative control. The CypA shRNA sequence used for this study was as follows: 5'CCACCGTGTTCTTCGACATTGCCGTCGAC3'. M055, KKU100, and MMNK1 cells were transiently transfected with pCMV6-XL5-CypA (OX-CypA) for CypA overexpression or pCMV6-XL5 empty vector (V). To do this, the CCA cells (2 × 10^5 ^cells) were seeded onto 6-well plate for 24 hours. The Lipofectamine™ 2000 and cDNA plasmids were diluted in Opti-MEM I medium and then combined at a ratio of 1:1. The cells were incubated with the diluted transfection complex in a total volume of 1 mL for 6 hours. Transfected cells were collected 48 hours after transfection and used for subsequent analysis.

### Stable cell line selection

Stable cells expressing shCypA were selected in M139 cells with retrovirus vector (OriGene). Briefly, Phoenix™ Ampho Cells (OriGene) were transfected with either shCypA or shV plasmids. Viral supernatants were collected and transduced into the parental M139 cells. Stable cell lines expressing CypA-shRNA (M139-shCypA) or negative control vector (M139-shV) were selected with the addition of 0.5 μg/mL puromycin into the medium; stable cell lines were cultured for at least 2 weeks before confirming the expression levels of CypA by real-time PCR and Western blot.

### Cell proliferation assay

The effects of CypA silence and overexpression on cell proliferation were determined by measuring cell viability using 3-(4,5-dimethylthiazol-2-yl)-5-(3-carboxymethoxyphenyl)-2-(4-sulfophenyl)-2H-tetrazolium (MTS) assay. Briefly, the cells transiently and stably transfected with shCypA or shV and transiently transfected with pCMV6-XL5-CypA or pCMV6-XL5 empty vector (OriGene) were seeded onto 96-well plates (2 × 10^3 ^cells/well) and serum-starved (0% FBS) for 24 hours. Then, they were incubated with RPMI 1640 medium supplemented with 2% FBS. Cell growth was assessed 1, 2, 3, 4, 5, and 6 days. Twenty μL of MTS reagent mixed with 100 μL serum free RPMI 1640 medium was added to each well and incubated at 37°C for 2 hours. Absorbance was recorded at 490 nm with an EL-800 universal microplate reader (Bio-Tek instruments).

Proliferation was also assessed in the presence of CsA. M139 cells were seeded onto a 96-well plate (5 × 10^3 ^cells/well) and serum-starved for 24 hours; then, RPMI 1640 medium containing 10% FBS and CsA (0, 0.01, 0.1, and 1 μg/mL) was added. Cells were incubated for 48 hours and subjected to MTS analysis as described above.

### Wound healing assay

The M139-shCypA and M139-shV cells were seeded onto 6-well plates (1.5 × 10^6 ^cells/well) and incubated in a humidified atmosphere of 5% CO_2 _at 37 °C for 24 hours. Wounds were generated on the surface of confluent monolayers using a sterile pipette tip, followed by incubation with RPMI 1640 medium supplemented with 0%, 2%, and 5% FBS. Pictures of a representative field were taken at 0 and 24 hours after scarification. Cell-free spaces were measured by ImageJ software and calculated as the wound width as a measure of cell migration. The wound width measured at 24 hours for each experiment was normalized to that measured at initial wound (0 hour), generating the relative migration distance.

### Animal model

The following animal work was approved by the Office for Protection from Research Risks (OPRR) and Animal Welfare Act guidelines under an animal protocol approved by Baylor College of Medicine Institutional Animal Care and Use Committee. The cells (3 × 10^6^) in a volume of 100 μL were inoculated into the subcutaneous tissue of the right flank of 5 to 6-week-old male nude mice (NCI Charles River); five animals per group were used. The tumor size was measured weekly using a digital caliper (VWR international). After 4 weeks, all mice were euthanized and tumors and organs (liver and lungs) were harvested; half of each specimen was fixed in buffered neutral formalin and the remaining half was snap-frozen in -80 °C.

### Statistical analysis

Experimental data were analyzed using SPSS 16.0 Windows Evaluation software (SPSS Inc., Chicago, IL). All quantitative data were expressed as mean ± SD. Two-tailed Student's t-test was used for comparison between two groups. Statistical significance was established at *P *< 0.05.

## Results

### CypA is overexpressed in liver fluke-associated CCA tissue specimens

**Figure 1:**
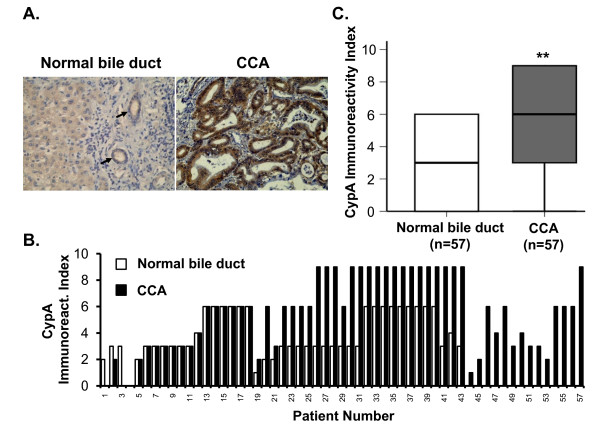
**Overexpression of CypA in CCA tissue specimens**. Immunohistochemistry of CypA expression in cancerous and cancer-adjacent normal bile duct was examined. (A) CypA immunostaining in one representative paired CCA case. Stronger staining for CypA (dark brown) is seen in tumor tissue compared with adjacent normal bile duct lining cells (arrows). (B) CypA immunoreactivity index in normal bile duct versus CCA for 57 paired CCA cases. The majority of the paired tissue specimens (39/57, 68%) demonstrated higher CypA expression in malignant areas than in adjacent normal bile duct areas. (C) The overall median of the CypA immunoreactivity index is significantly higher in CCA samples compared to the adjacent normal bile duct tissue (*P *< 0.05; n = 57).

Immunostaining for CypA was performed in 57 paraffin-embedded tissue specimens from patients with CCA in order to determine CypA protein expression patterns in CCA tumors in comparison with adjacent normal bile duct cells. Strongly positive staining for CypA was frequently observed in tumor specimens, while negative to moderately positive staining was observed in tumor-adjacent normal bile duct lining cells (Figure 1A). From these data, a semi-quantitative CypA immunoreactivity index was generated (Figure 1B). In the 57 tissue specimens, 68.4% (39 of 57) of the specimens had a higher CypA immunoreactivity index in the tumor tissue when compared with respective adjacent normal bile duct lining cells; 26.3% (15 of 57) demonstrated equal degrees of staining; and, only 5.2% (3 of 57) showed weakly positive staining in normal bile ducts and lack of staining in tumor areas. From the box plot in Figure 1C, The overall median CypA immunoreactivity index was significantly higher in CCA as compared with that of normal bile ducts (*P *< 0.05; n = 57). This upregulation of CypA in CCA tissues confirms its potential as a molecular target for CCA therapy.

### Endogenous expression of CypA in CCA cell lines correlates with cell growth phenotypes

**Figure 2:**
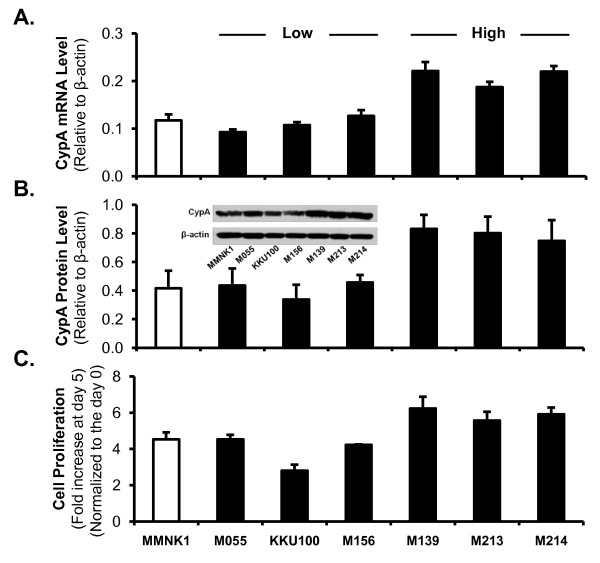
**Endogenous expression of CypA in CCA cell lines correlates with cell proliferation rates**. CypA expression was examined in 6 CCA cell lines and in a non-tumor human cholangiocyte cell line, MMNK1. When compared to MMNK1, three CCA cell lines (M055, KKU100, and M156) had relatively low CypA expression levels, and 3 cell lines (M139, M213, and M214) demonstrated relatively high CypA expression at both (A) mRNA and (B) protein levels as determined by real-time PCR and Western blot, respectively. (C) MTS cell proliferation assay demonstrates that low-CypA cells demonstrated lower proliferation rates than the high-CypA cells. MTS reading of each sample at the day 5 culture was normalized to that of its initial cell seeding at day 0, generating the fold increase.

We observed different endogenous CypA mRNA (Figure 2A) and protein (Figure 2B) levels among the 6 CCA cell lines studied. Comparing with a non-tumor cell line MMNK1, we divided the CCA cell lines into 2 groups. The first group had relatively high CypA expression (M139, M213 and M214); the second group had relatively low CypA expression (M055, KKU100 and M156). We further observed that, as shown in Figure 2C, the cells with low CypA levels demonstrated lower rates of cell proliferation than the cells displaying high CypA levels.

### Suppression of CypA reduces proliferation of M139 CCA cells *in vitro *and reduced proliferation may be mediated by reduction in PPIase activity

**Figure 3:**
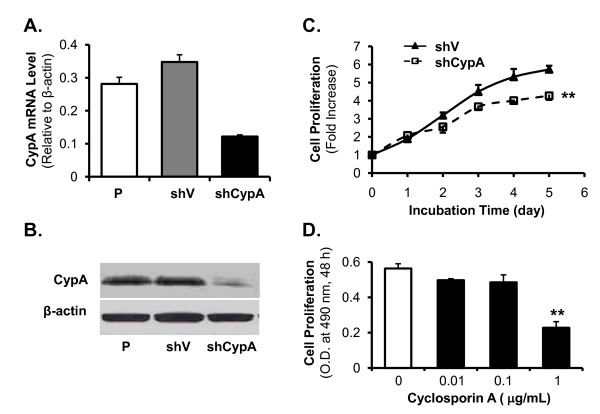
**Stable silence of CypA in M139 cells results in reduced cell proliferation**. Stable cell lines expressing shCypA (CypA silence, M139-shCypA) or control shRNA (empty vector, M139-shV) were generated. CypA silence was confirmed by (A) real-time PCR using β-actin as an internal control; and (B) Western blot. (C) Stable CypA silence led to reduced cell proliferation *in vitro*. Cell proliferation rates are shown as the fold increase, normalized to the rate at the time of seeding. (D) CsA treatment inhibits cell proliferation.

To further study the function of CypA on CCA cell growth, we determined the effects on cell proliferation of CypA silencing in M139, M213 and M214 cell lines (high-CypA cell lines) and CypA overexpression in M055 and KKU100 cell lines (low-CypA cell lines). For comparison, MMNK1 cells were included in both gene silence and overexpression studies. First, we performed CypA silence. The M139 cells were stably transfected with a shCypA (OriGene), and this led to about 65% and 76% CypA silence by real-time PCR (Figure 3A) and by Western blot analysis (Figure 3B), respectively. M139 cell transfected with a vector control shV showed no significant difference in CypA expression relative to parental M139 cells.

To determine whether the CypA silence affected cell proliferation, we performed MTS assays on M139-shCypA and M139-shV cells. As shown in Figure 3C, silencing of CypA in M139-shCypA cells was associated with a 25% reduction in cell proliferation by day 5, compared with that seen in M139-shV cells (Figure 3C, *P *< 0.05; n = 3).

To examine whether the PPIase activity of CypA could play a role in cell proliferation, M139 parental cells were incubated with CsA, an inhibitor of CypA PPIase, at concentrations of 0, 0.01, 0.1, and 1 μg/mL for 48 hours. Then, MTS assay was performed. Just as was seen with shRNA-mediated depletion of CypA, CsA treatment inhibited M139 cell proliferation in a dose-dependent fashion (Figure 3D).

### Suppression of CypA reduces migration of M139 CCA cells *in vitro*

**Figure 4:**
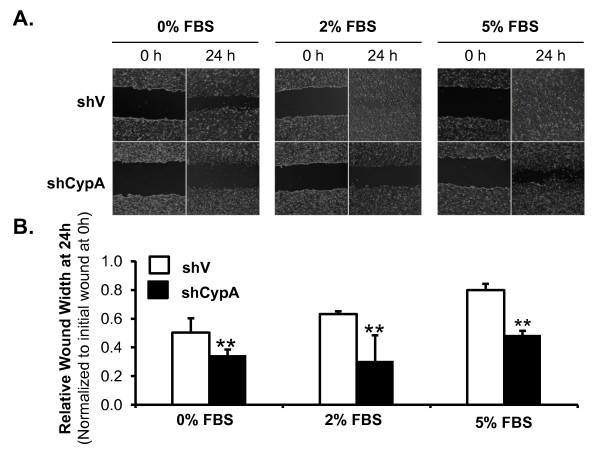
**Stable silence of CypA reduces wound healing ability, and thus migration ability of M139 cells *in vitro***. To measure cell migration potential, wound healing assay was carried out in M139-shV and M139-shCypA cells using RPMI 1640 medium supplemented with 0%, 2%, and 5% FBS. The wound width measured at 24 hours for each experiment was normalized to that measured at initial wound (0 hour), generating the relative migration distance. (A) Representative images taken from the same field at 0 and 24 hour. (B) Average relative wound width (relative migration distance) was compared among groups. Bar graphs represent means ± SD; n = 4, ** denotes *P *< 0.05 versus the control.

The effect of CypA silence on cell migration was also performed. A cell scratch test was carried out using M139-shCypA cells and the control M139-shV cells. Briefly, a sub-confluent monolayer of M139-shCypA and M139-shV cells was wounded at time 0, creating a cell-free space. Then, the cells were incubated in RPMI 1640 medium supplemented with different serum concentrations (0, 2 and 5% FBS) and monitored by phase-contrast microscope at 0 and 24 hours after wounding. The representative images taken from the same field at 0 and 24 hours are shown in Figure 4A. A smaller wound width following incubation, then, indicated a stronger migration capacity. The semiquantitative data showed that, for any serum concentration used, the M139-shCypA cells migrated into the cell free space significantly slower than the control cells (Figure 4B). Thus, CypA silence significantly reduces CCA cells' capacity for migration.

### The inhibitory effect of CypA silence on CCA cell proliferation is not limited to M139 cells

**Figure 5:**
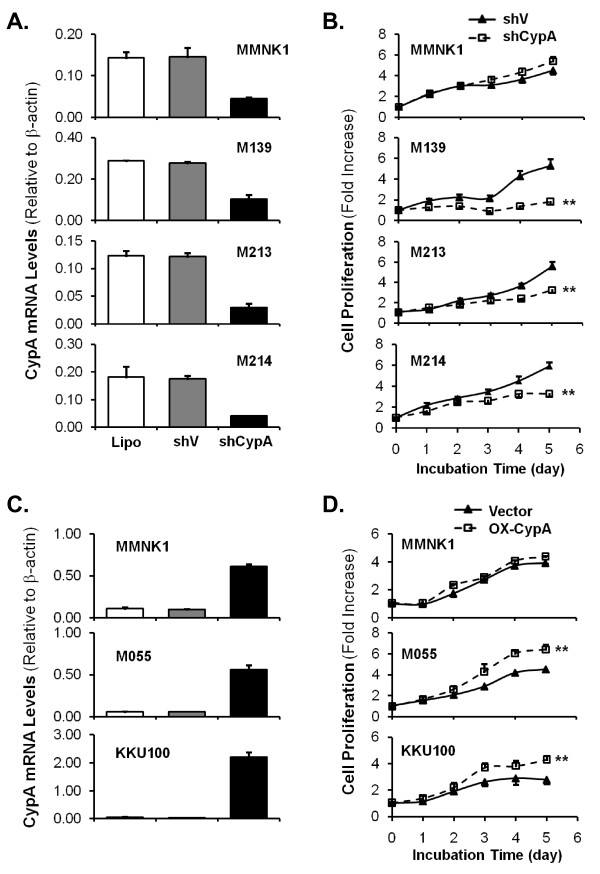
**Effects of transient silence and forced overexpression of CypA on cell proliferation of CCA cell lines and a non-tumor cholangiocyte cell line**. (A) CypA mRNA levels after transient silence of CypA in M139, M213, M214 CCA cell lines and MMNK1. (B) Transient silence of CypA reduces cell proliferation of CCA cells, but not MMNK1 cells *in vitro*. (C) CypA mRNA levels after transient overexpression of CypA for 48 hours. (D) Transient overexpression of CypA in CCA cell lines showed higher rate of cell proliferation compared to the control whereas MMNK1 cell proliferation was not affected by CypA overexpressed. Cell proliferation rate is shown as the fold increase compared to initial seeding. Data represent means ± SD of three independent experiments. ** denotes *P *< 0.05 versus the control.

Overall, we assessed the effects of CypA silence on CCA cell proliferation in three CCA cell lines displaying endogenously high CypA expression and in MMNK1 cells. M139, M213 and M214 CCA cell lines and MMNK1 cells were transiently transfected with either shCypA or scramble control shV using Lipofectamine™ 2000 as a transfection reagent. ShCypA transfection reduced CypA mRNA levels to 63%, 75%, 77%, and 70% in M139, M213, M214, and MMNK1 respectively, in comparison with levels in the cells transfected with only the shV control vector. No difference in CypA mRNA level was observed between the cells transfected with shV and cells transfected with only Lipofectamine™ 2000 (Figure 5A). MTS assay was again used to measure the cell proliferation. As shown in Figure 5B, silencing of CypA reduced cell proliferation in all three CCA cell lines but not in MMNK1. By day 5, proliferation was reduced by 65% in M139-shCypA cells, 41% in M213-shCypA cells, and 44% in M214-shCypA cells, compared with their respective vector controls (*P *< 0.05; n = 3). The cell proliferation of MMNK1 was not different between two groups.

### Forced overexpression of CypA increases CCA cell proliferation *in vitro*

We next examined the effects of CypA overexpression in MMNK1 and two CCA cell lines with relatively low endogenous CypA expression: M055 and KKU100. These cells were transfected with a pCMV6-XL5-CypA overexpression construct (OriGene) or with pCMV6-XL5 empty vector as a control. Overexpression of CypA in MMNK1 (MMNK1-OX-CypA), M055 (M055-OX-CypA), and KKU100 (KKU100-OX-CypA) cell lines was confirmed with real-time PCR (Figure 5C). When compared with their respective vector control cells (MMNK1-V, KKU100-V, and M055-V), CypA mRNA levels increased by 6-fold, 10-fold, and 55-fold in MMNK1-, M055-, and KKU100-OX-CypA cells, respectively, 48 hours after transfection.

Cell proliferation was assessed using MTS assay; this revealed that forced overexpression of CypA in CCA cells resulted in faster cell proliferation rates when compared with cells transfected with the empty vector. As shown in Figure 5D, at day 5 after seeding, cell proliferation rates for M055-OX-CypA and KKU100-OX-CypA were 30% and 35% higher than those of M055-V and KKU100-V, respectively (*P *< 0.05; n = 3). Again, CypA forced overexpression had no effect on cell proliferation of MMNK1 cells.

### CypA depletion attenuates ERK1/2 activity in M139 cells

**Figure 6:**
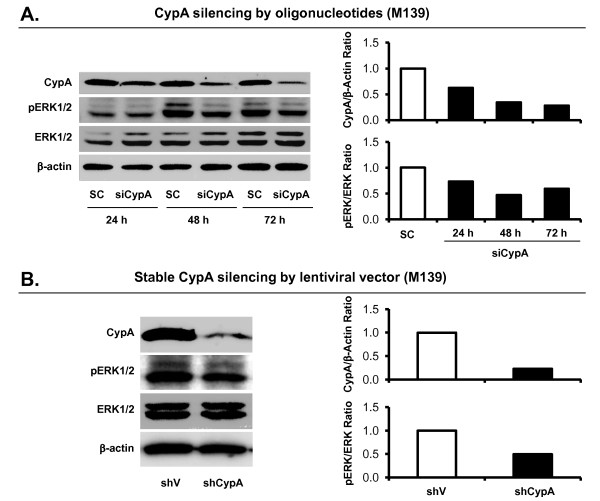
**CypA depletion attenuates ERK1/2 activity in M139 cells *in vitro***. ERK1/2 phosphorylation in CypA knockdown cells was determined by Western blot analysis. (A) Transient knockdown CypA decreased pERK1/2:ERK1/2 ratio in a time dependent fashion, and CypA protein level is positively correlated with ERK1/2 activity (phosphorylation). M139 cells (2 × 10^5 ^cells) were seeded in 6-well plate for 24 hours before being transfected with 100 pmole/mL siRNA against CypA or negative control siRNA for 6 hours before replacing with complete medium. Lipofectamine™ 2000 was used as a transfection reagent. Total proteins were collected at 24, 48, and 72 hours after transfection. (B) Stable knockdown CypA cells had lower ERK activity (phosphorylation) than the control cells. Protein samples were collected from M139-shV and M139-shCypA cells grown in complete medium for 24 hours. All samples were subjected to Western blot analysis for CypA, ERK1/2 and pERK1/2 detection, and b-actin was used as a loading control. Intensity of the immunoreactive bands was measured by ImageQuant TL software (GE Healthcare). Protein level of each sample was normalized to b-actin prior to comparison between samples. The pERK1/2:ERK1/2 ratio value reflects ERK1/2 activity.

The Ras-dependent ERK1/2 pathway plays a central role in controlling cell proliferation [30]. Several reports demonstrate that CypA-induced cancer cell proliferation is mediated by its membrane receptor CD147 and the ERK1/2 signaling pathway [14,17,18,22]. However, it is not clear whether these molecular mechanisms are involved in CCA. Small interfering RNA (siRNA) oligonucleotide duplexes were designed to specifically target a 21 base pair nucleotide sequence within CypA mRNA, corresponding to nucleotide position 334-354 (siCypA; QiaGen). Scrambled-sequence siRNA duplexes were used as a negative control (SC; Invitrogen). Transient suppression of CypA using siCypA in M139 cells inhibited the ERK1/2 phosphorylation in a time-dependent manner; the semi-quantitative analysis showed a positive correlation between the CypA protein level and phosphorylation of ERK1/2 protein (Figure 6A). Furthermore, the effect of CypA on the ERK1/2 activity was determined in stable CypA knockdown clones; ERK1/2 activity was substantially decreased in M139-shCypA cells compared with that in M139-shV cells (Figure 6B). Thus, CypA appears to be involved in the ERK1/2 signal transduction pathway.

### Suppression of CypA leads to slower tumor growth and decreased cell proliferation *in vivo*

**Figure 7:**
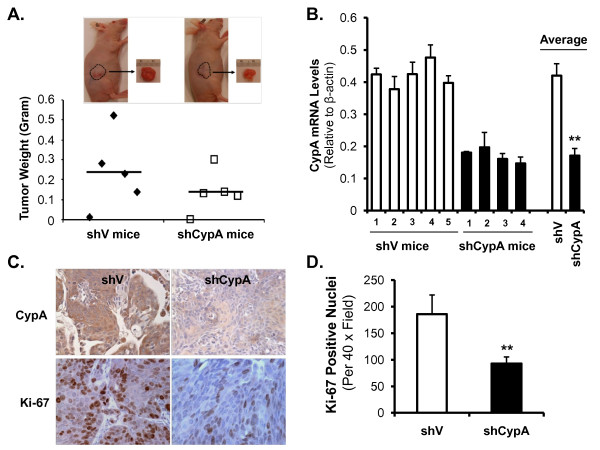
**Stable silence of CypA results in decreased tumor growth and cell proliferation in a nude mouse model of subcutaneous (s.c.) xenograft**. (A) Tumor weight (in grams). Insets: a representative mouse picture and s.c. tumor mass from each group. (B) Tumor tissues from M139-shCypA inoculated mice had significant lower levels of CypA-mRNA than those from M139-shV mice. The stability of CypA silencing in tumor tissues derived from M139-shCypA cells was confirmed by real-time PCR. (C) Immunohistochemical staining for CypA and Ki-67. (D) Quantification of Ki-67 immunohistochemical staining. Three tumors per group were analyzed and Ki-67 positive nuclei from four random high power (40×) fields were counted for each tumor tissue. Lower frequency of Ki-67 positive cells is shown in the tissue from M139-shCypA mice compared with that of Ki-67 positive cells from M139-shV mice (** *P < 0.05*).

The effect of CypA silence on tumor growth was analyzed *in vivo *using an immunodeficient nude mouse model (n = 5 for each group). Four weeks after s.c. implantation, tumors were removed, weighed and processed for further histological and immunohistochemical analysis. Tumors from mice injected with M139-shCypA cells were smaller in size and weight compared with those from mice treated with M139-shV cells; there was a 43% reduction in weight in the M139-shCypA tumors (0.138 ± 0.107 g vs 0.240 ± 0.187 g, Figure 7A). Furthermore, we confirmed that CypA silence persisted in the M139-shCypA cell line-derived tumors by real-time PCR and immunostaining for CypA (Figure 7B and 7C). We also determined that tumors from M139-shCypA injected mice demonstrated reduced cell proliferation, as indicated by fewer Ki-67 positive staining nuclei than observed in vector control tumors (*P *< 0.05; Figure 7C and 7D). These results indicate that CypA silence had a significant effect on tumor growth *in vivo*.

## Discussions

CCA is refractory to conventional chemotherapy and has an extremely poor prognosis; therefore, the identification of novel molecular targets for the diagnosis, prognosis, and treatment of CCA is a critical task for investigators. There are numerous studies suggesting a role for CypA in tumorigenesis and progression of human cancer, and overexpression of CypA in various tumor tissues has been shown to both confer growth advantage and correlate with poor clinical outcomes [31]. At present, however, little is known about the expression of CypA in CCA tissue specimens. Using expressed sequence tags, CypA was reported to be upregulated in intrahepatic CCA compared with normal liver tissues [32]. In this study, however, using immunohistochemistry, we provide evidence for the first time that CypA is localized in tumor bile duct epithelia and upregulated in CCA tumor tissue samples as compared with tumor-adjacent normal bile duct lining cells. In addition, we determined that normal bile ducts along the margins of tumor areas, as well as proliferative normal bile ducts, frequently display stronger positive staining for CypA than seen in more geographically distant points along the bile ducts. These observations suggest that "normal" bile duct near the tumor margin and proliferative bile duct are probably not normal molecularly, regardless of their histological appearance. Furthermore, we observed that normal liver tissues in all specimens either lack CypA staining or demonstrate weak background staining, which implies that using CypA as a molecular target for CCA treatment may be feasible.

In the present study, we demonstrated that reducing PPIase activity of CypA by CsA also significantly decreased proliferation of CCA cell lines. CsA is an active compound which specifically inhibits the activity of cyclophilin family, of which CypA is the major and tightest-binding CsA target identified to date [33,34]. It binds with sub-nanomolar affinity to CypA via contact within the hydrophobic pocket of CypA and inhibits its PPIase activity [35]. As an inhibitor of CypA PPIase activity, either as a single agent or in combination with other chemotherapeutic drugs, CsA induced antiproliferative and proapoptotic effects at clinically achievable levels [36-38]. In addition, CsA and its non-immunosuppressive derivative NIM811 were shown to have similar capability of inducing apoptosis of malignant melanoma cells *in vitro *and *in vivo *studies [39]. Thus, targeting CypA using CsA as a chemosensitizer, CsA derivatives without immunosuppressant activity, and novel inhibitors of CypA may likely improve cancer treatment including CCA.

Importantly, our *in vitro *cell proliferation studies clearly showed that CypA positively regulates CCA cell proliferation. CypA silence significantly reduces cell proliferation in M139, M213, and M214 cells, while forced overexpression of CypA in two other CCA cell lines, M055 and KKU100, had the converse effect; M055 and KKU100 cells overexpressing CypA demonstrated significantly higher rates of cell proliferation than the respective vector control cells, which had relatively low endogenous CypA expression. On the other hand, it seems that CypA does not play significant role in controlling cell proliferation of a non-tumor cholangiocyte cells as manipulating CypA levels by gene knockdown and overexpression experiments had no effects on MMNK1 cell proliferation. A previous study reported that CypA is a key factor for vascular smooth muscle cell proliferation; in contrast, it induces endothelial cell apoptosis [40]. These different results reflect multiple functions of CypA in different cell types depending on intracellular contexts, binding partners as well as its positive/negative regulators.

The functional role of CypA in cancer cell differentiation is not clear. Our data indicate that malignant phenotypes, including cell proliferation, migration, and drug resistance properties, of CCA cells are not necessarily correlated with cell differentiation. For example, M055 cell line, which derived from poorly differentiated cholangiocarcinoma is the slowest growing cell compared with other CCA cell lines, and it is also most susceptible to apoptosis. In the present study, we observed that the CypA level in parental CCA cells agreed with the cell proliferation rate of each cell line regardless of the degree of cell differentiation, implying that CypA may be one of the molecules that play an important role in CCA cell proliferation.

Tumor weight measured in shCypA-inoculated mice was higher than that of shV-inoculated mice; cell proliferation in using Ki-67 staining clearly showed reduced cell proliferation rates in CypA silence tumors (93 ± 12.5) compared with tumors expressing scramble control vector (186 ± 36.0, *P *< 0.05). These results were consistent with previous studies in endometrial carcinoma [25], osteosarcoma [23] and lung cancer [41].

## Conclusions

**Figure 8:**
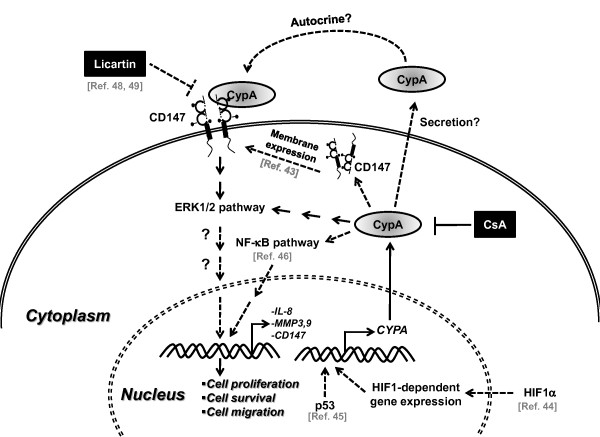
**A schematic representation of a proposed mechanism involved in CypA-mediated cell proliferation in CCA**. CypA is upregulated in CCA cells, and several carcinogenic factors such as HIF-1a and p53 may regulate CypA expression. Unusual high CypA protein level may lead to sustained activation of ERK1/2 and NF-kB through direct or indirect mechanisms, which in turn activate cell survival, proliferation, and tumor progression. CypA may be secreted from the cells and act as an autocrine/paracrine growth factor via interacting with its membrane receptor CD147. Using specific inhibitor against CypA or against CypA/CD147 binding may inhibit CCA cell proliferation and tumor progression.

We demonstrated that CypA is upregulated in human CCA samples; CypA expression correlates with a malignant cell growth phenotype in CCA cell lines; inhibiting CypA expression reduces CCA cell proliferation and migration *in vitro*, with the effects on proliferation likely mediated by reduced enzymatic activity and that the ERK1/2 pathway may be involved; overexpression of CypA enhances cell proliferation; and, finally, that suppression of CypA reduces tumor size and cell proliferation *in vivo*. As an autocrine/paracrine mechanism, extracellular CypA could activate the ERK1/2 pathway via CD147. Reduction of intracellular CypA levels via shRNA may lead to insufficient extracellular CypA levels, which in turn reduce cell proliferation. On the other hand, intracellular CypA itself may mediate gene expression or act as a chaperone for its membrane receptor CD147 [22,42,43]. Based on our results and other published data, we schematically show the possible mechanisms of CypA-mediated cell proliferation in CCA (Figure 8). CypA is upregulated in CCA cells, and several carcinogenic factors including cell stress response genes, HIF-1a and activated p53, may increase CypA expression [44,45]; the high CypA levels could directly or indirectly activate the ERK1/2 signaling pathway and NF-κB pathway [46], which in turn mediate gene transcription of interleukin (IL)-8, and matrix metalloproteinase (MMP) 3 and 9 [47]. CypA may act as a chaperone protein to facilitate CD147 membrane expression and stabilization; and CypA may be secreted by CCA cells and act as an autocrine/paracrine molecule via CD147, thereby activating the ERK1/2 pathway and stimulating cell proliferation. In the future, detailed studies on molecular mechanisms by which CypA affects cancer progress in CCA will help us to understand more about this devastating cancer and develop new chemotherapeutic agents. For examples, specific inhibitor of CypA without immunosuppressive effect and a CD147 inhibitor, Licartin, should be included in future investigations [48,49]. Presently, we are engaged in an ongoing project focused on the roles of extracellular CypA and its trans-membrane receptor CD147 as well as the subsequent downstream signaling pathway; we hope to gain new insight into the molecular mechanisms by which CypA promotes CCA cell proliferation. These studies may be further extended into an investigation of the role of CypA in the regulation of CCA cell growth in the complex tumor environment. Finally, treatment with non-immunosuppressive CsA derivatives, novel CypA inhibitors and/or inhibitors of CypA-mediated signaling may lead to better treatment outcomes for CCA patients with high CypA expression.

## List of Abbreviations

CypA: cyclophilin A; CCA: cholangiocarcinoma; RT-PCR: reverse transcriptase-polymerase chain reaction; MTS: 3-(4,5-dimethylthiazol-2-yl)-5-(3-carboxymethoxyphenyl)-2-(4-sulfophenyl)-2H-tetrazolium assay; PPIase: peptidylprolyl *cis-trans *isomerase; ERK1/2: ras-dependent extracellular signal-regulated kinase-1 and -2; pERK1/2: phosphorylated ERK1/2; kDa: kiloDalton; CsA: cyclosporin A; Ig: immunoglobulin; shRNA: short hairpin RNA; FBS: fetal bovine serum albumin; EDTA: ethylenediaminetetraacetic acid; HRP: horseradish peroxidase; PBS: phosphate buffered saline; DAB: 3, 3'-diaminobenzindine tetrahydrochloride; cDNA: complementary DNA; PPIA: peptidylprolyl *cis-trans *isomerase A; RIPA: radio immuno-precipitation assay; SDS: sodium dodecyl sulfate; PVDF: polyvinylidene difluoride; TBS: tris-buffered saline; ECL: enhanced chemiluminescence; GFP: green fluorescent protein; SD: standard deviation; siRNA: small interfering RNA; SC: scrambled siRNA control; IL-8: interleukin-8; MMP: matrix metalloproteinase; NF-κB: Nuclear Factor Kappa Beta; HIF1-α: hypoxia-inducible transcription factor 1 alpha

## Authors' contributions

SO, SW, CW, KS, QY and CC designed research; SO performed research; SO, SMW, SW, CW, KS, QY and CC analyzed data; and SO, SMW, SW and CC wrote the paper. All authors read and approved the final manuscript.

## Competing interests

The authors declare that they have no competing interests.
